# Supplementary material for: Bypassing the Kohn-Sham equations with machine learning
Source: Nat Commun. 2017 Oct 11;8:872. doi: 10.1038/s41467-017-00839-3 (PMC5636838; doi:10.1038/s41467-017-00839-3)
Supplement: Supplementary file 1 — Supplementary Information [file 41467_2017_839_MOESM1_ESM.pdf]

Description of Supplementary Files

File Name: Supplementary Information
Description: Supplementary Figures, Supplementary Table, Supplementary Notes, Supplementary Methods and Supplementary References

File Name: Peer Review File
Description:

SUPPLEMENTARY METHODS

Kernel Ridge Regression

Kernel Ridge Regression[1, 2] (KRR) is a machine learning method for regression. We introduce the method for abstract training points $(\mathbf{x}_i, \mathbf{y}_i)$, i.e. features $\mathbf{x}_1, \dots \mathbf{x}_M \in \mathbb{R}^d$ and associated labels $\mathbf{Y} = (\mathbf{y}_1, \dots, \mathbf{y}_M)^T \in \mathbb{R}^M$ and describe the actual models used in the main text afterwards. We want to model a function $f : \mathbb{R}^d \to \mathbb{R}$ that maps from features to labels. This model should not be 'learned by heart' but perform well on unseen data (i.e. generalize). We first restrict the set of possible functions to the reproducing kernel Hilbert space (RKHS) $\mathcal{H}$ on the space of discretized densities that is induced by the Gaussian kernel function

$$k(\mathbf{x}, \mathbf{x}') = \exp\left(-\frac{||\mathbf{x} - \mathbf{x}'||^2}{2\sigma^2}\right). \tag{1}$$

The restriction is very mild and rather technical; more interesting is the choice of the kernel function which determines the scalar product (and thus the norm) of the RKHS. Leaving rigor aside, the Gaussian kernel induces an RKHS norm $||f||_{\mathcal{H}}$ that is smaller for simpler, smoother functions and higher for more complicated, oscillating functions. We minimize the empirical risk functional

$$\mathcal{C}(f) = \sum_{i=1}^{M} |\mathbf{y}_i - f(\mathbf{x}_i)|^2 + \lambda \|f\|^2_{\mathcal{H}} \tag{2}$$

that defines a trade-off between error on the training points and smoothness of the function controlled by the hyper-parameter λ.

The representer theorem[3] allows us to assume that the solution to Eq. 2 is given by a linear combination of kernel functions $f = \sum_{i=1}^{M} \boldsymbol{\alpha}_i k(\mathbf{x}_i, \cdot)$. It now suffices to solve

$$\mathcal{C}(\boldsymbol{\alpha}) = \sum_{i=1}^{M} |\mathbf{y}_i - f(\mathbf{x}_i)|^2 + \lambda \|f\|^2_{\mathcal{H}} \tag{3}$$

$$= \sum_{i=1}^{M} |\mathbf{y}_i - f(\mathbf{x}_i)|^2 + \lambda \boldsymbol{\alpha}^{\intercal} \mathbf{K} \boldsymbol{\alpha}, \tag{4}$$

where $\mathbf{K}_{ij} = k(\mathbf{x}_i, \mathbf{x}_j)$ is the kernel matrix. The solution is given by

$$\boldsymbol{\alpha} = (\mathbf{K} + \lambda \mathbf{I})^{-1} \mathbf{Y}. \tag{5}$$

Note that all model parameters and hyper-parameters are estimated on the training set; the hyper-parameter choice makes use of standard cross-validation procedures (see Hansen *et al.* [4]). Once the model is fixed after training, it is applied unchanged out-of-sample.

We use this method for various maps:

Non-interacting kinetic energy functional ($T_{\mathrm{s}}^{\mathrm{ML}}[n]$, 1-D). The training points are given by pairs of densities and associated kinetic energies. We discretize the densities and use them in vectorial form, i.e. $\mathbf{n} \in \mathbb{R}^G$. Thus, the functional $\mathcal{L}^2 \to \mathbb{R}$ is modeled as a function $\mathbb{R}^{\mathbb{G}} \to \mathbb{R}$

ML-OF map (1-D). The training points are given by pairs of discretized 1-D box potentials and associated total energies.

ML-KS map (3-D). The training points are given by pairs of discretized Gaussians potentials (as described in the main text) and total energies.

Total energy functional ($E^{\mathrm{ML}}[n]$, 3-D). The training points are given by pairs of densities in basis function representation (see below) and associated total energies. Just as for $T_{\mathrm{s}}^{\mathrm{ML}}$, this functional is modeled as a function.

ML Hohenberg-Kohn map

The basis representation for the densities is given by

$$n(x) = \sum_{l=1}^{L} \mathbf{u}^{(l)} \phi_l(x), \tag{6}$$

where ϕ_l are the L basis functions. We introduce some notation and write the density in grid representation as $\mathbf{n}$, and its basis coefficients as $\mathbf{u}$. We can then write the HK map model as

$$n^{\mathrm{ML}}[v](x) = \sum_{l=1}^{L} u^{(l)}[v] \phi_l(x), \tag{7}$$

where the L basis function coefficients are regular KRR models,

$$u^{(l)}[v] = \sum_{i=1}^{M} \boldsymbol{\beta}_i^{(l)} k(v, v_i), \tag{8}$$

of external potentials v with a Gaussian kernel function. The contribution of the error to the cost function can be formulated as

$$e(\boldsymbol{\beta}) = \sum_{i=1}^{M} \| n_i - n^{\mathrm{ML}}[v_i] \|_{\mathcal{L}_2}^2 \tag{9}$$

$$= \sum_{i=1}^{M} \left\| n_i - \sum_{l=1}^{L} \sum_{j=1}^{M} \beta_j^{(l)} k(v_i, v_j) \phi_l \right\|_{\mathcal{L}_2}, \tag{10}$$

with the $\mathcal{L}_2$ norm. We write this cost function in terms of basis function coefficients. This can be viewed as projecting the inside of the norm on each basis function. Assuming orthogonality of the basis functions yields

$$e(\boldsymbol{\beta}) = \sum_{i=1}^{M} \sum_{l=1}^{L} \left| \mathbf{u}_i^{(l)} - \sum_{j=1}^{M} \boldsymbol{\beta}_j^{(l)} k(v_i, v_j) \right|^2 . \tag{11}$$

where $\mathbf{u}_i^{(l)} = \langle n_i, \phi_l \rangle$ is the l-th basis function coefficient of the i-th training density, as defined in Eq. 6 if orthogonality is satisfied. After reordering the sums over i and l, we view each l independently and solve analogously to regular KRR

$$\boldsymbol{\beta}^{(l)} = \left(\mathbf{K}_{\boldsymbol{\sigma}^{(l)}} + \boldsymbol{\lambda}^{(l)} \mathbf{I} \right)^{-1} \mathbf{u}^{(l)}, \quad l = 1, \ldots, L \tag{12}$$

where, for each basis function l, $\boldsymbol{\lambda}^{(l)}$ is a regularization parameter, $\mathbf{K}_{\boldsymbol{\sigma}^{(l)}}$ is a Gaussian kernel with kernel width $\boldsymbol{\sigma}^{(l)}$. The $\boldsymbol{\lambda}^{(l)}$ and $\boldsymbol{\sigma}^{(l)}$ can be chosen individually for each basis function via independent cross-validation (see [4, 5]).

SUPPLEMENTARY NOTE 1: BASIS FUNCTIONS

Fourier basis. We define the basis as

$$\phi_l(x) = \begin{cases} \cos\{2\pi x(l-1)/2\}, & l \text{ odd} \\ \sin\{2\pi xl/2\}, & l \text{ even} \end{cases} \quad l = 1, \ldots, L. \tag{13}$$

We transform the density efficiently via the discrete Fourier transform

$$\mathbf{u}_i^{(l)} = \sum_{m=1}^{G} n_i(x_m)\phi_l(x_m). \tag{14}$$

The back-projection is written as

$$n^{\mathrm{ML}}(x) = \sum_{l=1}^{L} \mathbf{u}^{(l)}\phi_l(x). \tag{15}$$

KPCA basis. We define the basis as:

$$\boldsymbol{\phi}_l^{\mathrm{KPCA}} = \sum_{j=1}^{M} \mathbf{p}_j^{(l)}\Phi(n_j). \tag{16}$$

The parameters $\mathbf{p}_j^{(l)}$ are found by eigen-decomposition of the Kernel matrix. The KCPA basis coefficients are given by

$$\mathbf{u}_i^{(l)} = \langle \Phi(n_i), \phi_l^{\mathrm{KPCA}} \rangle = \sum_{j=1}^{M} \mathbf{p}_j^{(l)} k(n_j, n_i) \tag{17}$$

with kernel map Φ. The back-projection for KPCA is not trivial but several solutions exist. We follow Bakir *et al.* [6] and learn the back-projection map.

SUPPLEMENTARY NOTE 2: GRADIENT DESCENT ISSUES

There are two ways to remedy problems of the gradient descent procedure: First, the gradient descent step can be "de-noised" by projecting the gradient onto the data manifold and thus removing the noisy directions. Secondly, the directions outside of the data manifold can be removed in a preprocessing step to get rid of the influence of the noisy directions on the gradient completely. Both methods yield similar results.

Several approaches exist for describing and projecting onto the data manifold. Common to each approach is the idea to find principle components and to project on those in which direction the densities have largest variance. Best results are reported [7] by using Kernel Principle Component Analysis[8] (KPCA), a non-linear generalization of PCA.

There are three issues with the assumed gradient-based approaches: First, the correct choice of the number of (K)PCA components K has to be made. It is generally possible to view it as a hyper-parameter and find the optimal K via cross-validation. However, we can not choose fractional Ks. One K might be not enough and $K + 1$ too much information. Second, the data points only lie in a bounded region of a manifold that can be described via PCA components. It is still possible for the gradient descent to walk outside this bounded region toward a point where the model has no information and thus the gradients become inaccurate. A (K)PCA method that only accesses the scalar products between points in the data set can not solve this[9]. Third, it might not be possible to find a suitable pre-image for a ground-state density given by (K)PCA coefficients[10].

SUPPLEMENTARY NOTE 3: MOLECULAR DATASETS

For our 3-D DFT calculations in Quantum Espresso[11], we center a water molecule in a cubic cell and converge three variables: the kinetic energy cutoff for wavefunctions `ecutwfc` in steps of 10 Ry, the kinetic energy cutoff for charge density and potential `ecutrho` in steps of 40 Ry, and the cell dimension `celldm` in steps of 1 bohr. We increase parameters until increasing any parameter does not change the equilibrium position total energy by more than 0.01 kcal/mol for H_2O. We end up with `ecutwfc` of 90 Ry, `ecutrho` of 360 Ry, and `celldm` of 20 bohr, which are used for all other molecules in this work.

The extent of the dataset for H_2O is visualized in Supplementary Fig. 1. In this case, conformers were generated from random displacements from the optimized geometry.

For benzene and ethane, conformers were generated from isothermal molecular dynamics (MD) trajectories. The range of atomic positions from combined 1 ns 300 K and 350 K trajectories is shown in Supplementary Fig. 2 for benzene and Supplementary Fig. 3 for ethane after snapshots are aligned to a reference molecule.

For malonaldehyde, the classical MD trajectories include 0.5 ns for each tautomer at 300 K and 350 K. Resulting conformers used to create the K-means sampled training set are shown as red points in Fig. 6 of the main text. The test set to evaluate the energy error is taken from an ab initio MD trajectory at 300 K. The ML-HK model is also used to generate an MD trajectory using a finite difference method to calculate atomic forces at each timestep. A displacement of $\epsilon = 0.001$ Å was chosen to maintain energy conservation during the MD simulation using the Atomistic Simulation Environment (ASE) [12]. A Langevin thermostat with a friction coefficient of 0.01 atomic units (0.413 fs^{-1}) was selected to reproduce the fluctuations in atomic coordinates observed for the trajectory generated using DFT (see Supplementary Table 1). The ML-HK model generates a trajectory that visits molecular conformations at the extremes of the classically-sampled training set, with predicted energies lower than those calculated using DFT directly (see Supplementary Fig. 4). The largest predicted energy errors are observed for these high-energy conformers. However, the calculated forces are sufficiently large to bring the atoms back toward their equilibrium positions, resulting in a stable molecular trajectory.

SUPPLEMENTARY NOTE 4: SAMPLING

For H_2, since there is only one atomic distance to adjust, we take the M equi-distant points in the parameter range and for each of these points select the training point that is closest.

For larger molecules with more parameters (H_2O, Benzene, Ethane, Malonaldehyde) we also want to cover the conformer space in a way that all conformers are relatively close to at least one training point.

Assuming $\mathbf{p}_i$ are the parameters of conformer i and $i \in \tilde{\mathbf{P}}_j$ if and only if $\tilde{\mathbf{p}}_j$ is closest to $\mathbf{p}_i$, we want to find $\tilde{\mathbf{p}}_j$, $j = 1 \dots M$ that minimize

$$\sum_{j=1}^{M} \sum_{i \in \tilde{\mathbf{P}}_j} ||\tilde{\mathbf{p}}_j - \mathbf{p}_i||^2. \tag{18}$$

K-means[13] solves this problem for continuous $\tilde{\mathbf{p}}_j$. However, since K-means returns only locally optimal solutions, we rerun the algorithm 50 times and select the solution which minimizes Eq. 18. We choose the points $\mathbf{p}_i$ closest to each $\tilde{\mathbf{p}}_j$ as training points.

SUPPLEMENTARY NOTE 5: LOGIC OF DENSITY FUNCTIONAL THEORY (DFT)

Within the Born-Oppenheimer approximation in non-relativistic quantum mechanics, and using atomic units, the Hohenberg-Kohn paper[14] laid the theoretical framework of all modern DFT. The first statement is that the mapping

$$v(\mathbf{r}) \longleftrightarrow n(\mathbf{r}) \tag{19}$$

is one-to-one, i.e., at most one potential can give rise to a given ground-state density, even in a quantum many-body problem, for given interaction among particles and statistics (i.e., fermions or bosons). A follow-up claim is that the ground-state energy of an electronic system can be found from

$$E[v] = \min_n \left\{ F[n] + \int d^3r \, n(\mathbf{r}) v(\mathbf{r}) \right\} \tag{20}$$

where $F[n]$ is a density functional containing all many-body effects. The minimizing density is the solution to the Euler equation:

$$\frac{\delta F}{\delta n(\mathbf{r})} + v(\mathbf{r}) = \text{const} \tag{21}$$

It is the direct map between densities and potentials that we machine-learn in this paper. We call it the HK density map, $n[v](\mathbf{r})$.

The KS scheme avoids direct approximation of F by imagining a fictitious system of non-interacting electrons with the same density as the real one[15]. The KS equations are:

$$\left\{ -\frac{1}{2}\nabla^2 + v_\mathrm{s}(\mathbf{r}) \right\} \phi_i(\mathbf{r}) = \boldsymbol{\epsilon}_i \phi_i(\mathbf{r}) \tag{22}$$

where $\boldsymbol{\epsilon}_i$ are the KS eigenvalues and ϕ_i the KS orbitals.

$$v_\mathrm{s}(\mathbf{r}) = v(\mathbf{r}) + v_\mathrm{H}(\mathbf{r}) + v_\mathrm{XC}(\mathbf{r}) \tag{23}$$

where $v_\mathrm{H}(\mathbf{r})$ is the Hartree potential and $v_\mathrm{XC}(\mathbf{r})$ is the exchange-correlation potential. The true energy of the system is then reconstructed from the self-consistent density $n(\mathbf{r}) = \sum_i |\phi_i(\mathbf{r})|^2$ via

$$E[n] = T_\mathrm{s}[n] + U[n] + \int d^3r \, n(\mathbf{r}) \, v(\mathbf{r}) + E_\mathrm{XC}[n] \tag{24}$$

where $T_\mathrm{s}[n]$ is the kinetic energy of the non-interacting electrons and $U[n]$ is the Hartree energy. $E_\mathrm{XC}[n]$ is the exchange-correlation (XC) energy and implicitly defined by Eq. 24. Most calculations[16] use simple approximations that depend only on the density and its gradient to determine E_XC, called generalized gradient approximations, or replace a fixed fraction of the approximate exchange with the exact exchange from a Hartree-Fock calculation (called a hybrid). Requiring the XC potential to be the functional derivative of E_XC ensures that the self-consistent solution of Eq. 22 minimizes the energy of Eq. 24 for the given $v(\mathbf{r})$ and $E_\mathrm{XC}[n]$.

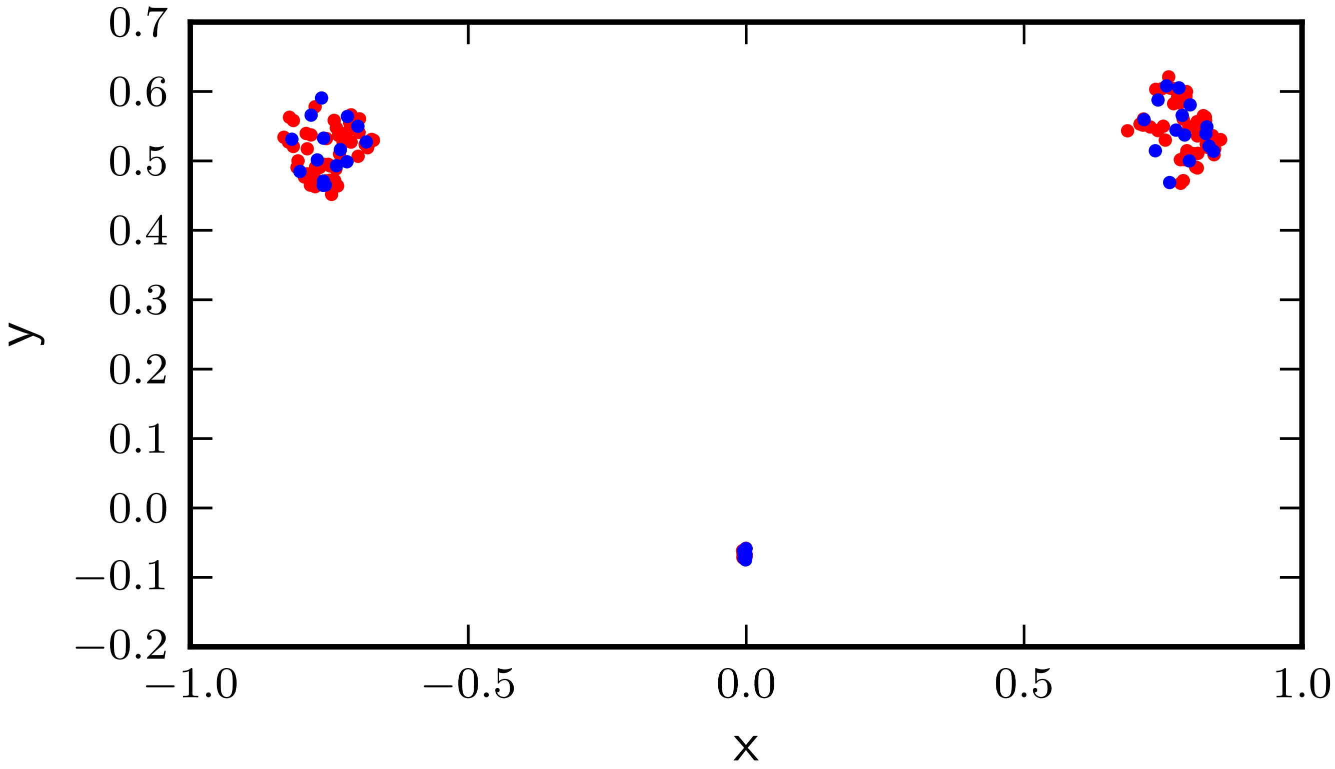

Supplementary Figure 1. **The extent of the H_2O dataset.** The figure shows the atom coordinates in angstrom. Blue are atoms from 15 training points, red from 50 test points.

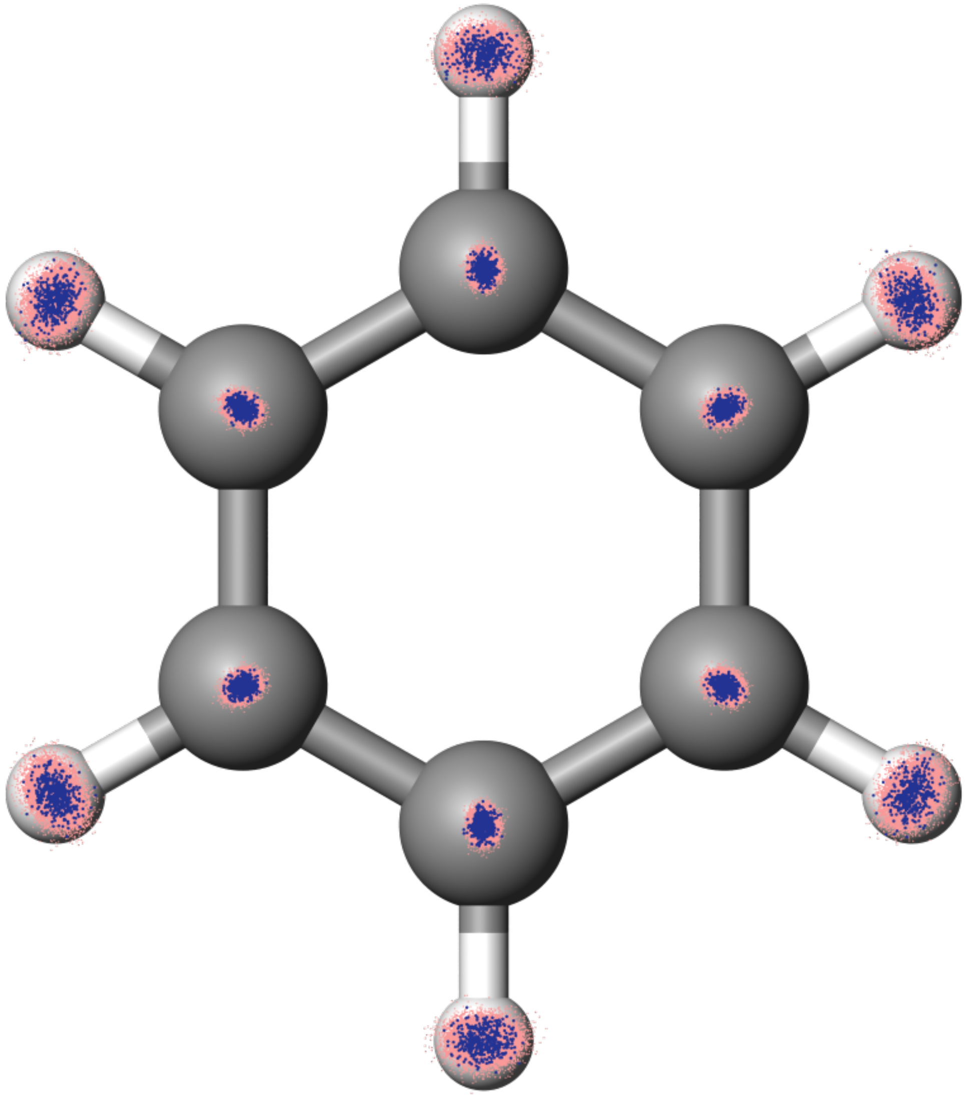

Supplementary Figure 2. **The extent of the benzene conformers.** The conformers generated by MD are displayed in red. K-means sampling is used to select 2,000 representative points. Test points from an independent trajectory are in blue and are offset from the molecular plane for clarity.

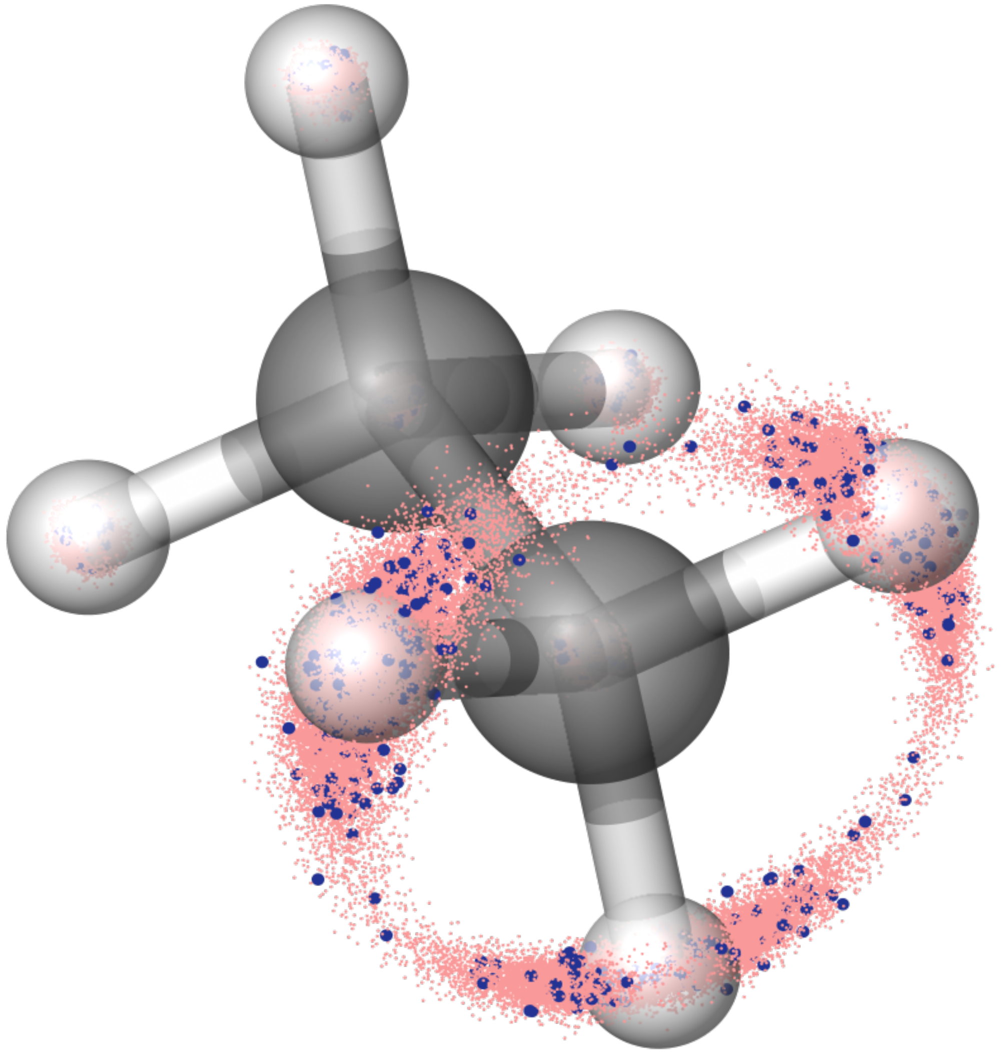

Supplementary Figure 3. **The extent of the ethane conformers.** The conformers generated by MD are displayed in red. K-means sampling is used to select 2,000 representative points. Test points from an independent trajectory are in blue.

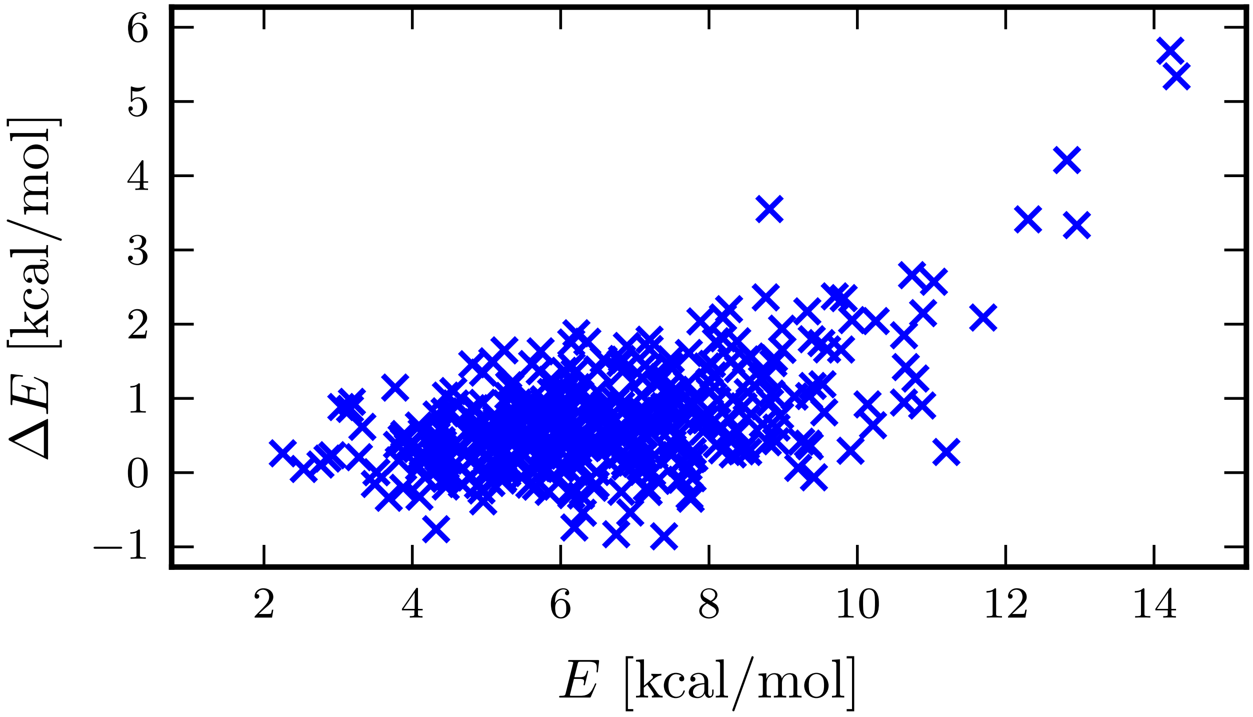

Supplementary Figure 4. **Total energy errors from ML-HK generated trajectory snapshots.** The largest energy errors are for high-energy conformations at the extremes of the classical training set coordinates.

MD Trajectory	Atom Type			
	C	O	H (-CH)	H (-OH)
DFT	0.052	0.076	0.166	0.289
ML-HK	0.051	0.094	0.171	0.242

Supplementary Table 1. **Difference between DFT and ML-HK sampling of malonaldehyde configurations.** Root mean squared deviations (Å) for malonaldehyde during 2 ps MD simulations relative to the average coordinates of the two optimized enol tautomers.

SUPPLEMENTARY REFERENCES

[1] Hastie, T., Tibshirani, R. & Friedman, J. *The Elements of Statistical Learning - Data Mining, Inference, and Prediction.* Springer Series in Statistics (Springer, 2009), 2nd edn.

[2] Vu, K., Snyder, J. C., Li, L., Rupp, M., Chen, B. F., Khelif, T., Müller, K.-R. & Burke, K. Understanding kernel ridge regression: Common behaviors from simple functions to density functionals. *Int. J. Quantum Chem.* **115**, 1115–1128 (2015).

[3] Schölkopf, B., Herbrich, R. & Smola, A. J. *A Generalized Representer Theorem*, 416–426 (Springer Berlin Heidelberg, Berlin, Heidelberg, 2001).

[4] Hansen, K., Montavon, G., Biegler, F., Fazli, S., Rupp, M., Scheffler, M., von Lilienfeld, O. A., Tkatchenko, A. & Müller, K.-R. Assessment and validation of machine learning methods for predicting molecular atomization energies. *J. Chem. Theory Comput.* **9**, 3404–3419 (2013).

[5] Müller, K.-R., Mika, S., Rätsch, G., Tsuda, K. & Schölkopf, B. An introduction to kernel-based learning algorithms. *IEEE Trans. Neural Netw.* **12**, 181–201 (2001).

[6] Bakir, G. H., Weston, J. & Schölkopf, B. Learning to find pre-images. In Thrun, S., Saul, L. K. & Schölkopf, B. (eds.) *Advances in Neural Information Processing Systems*, vol. 16, 449–456 (MIT Press, 2004).

[7] Snyder, J. C., Rupp, M., Müller, K.-R. & Burke, K. Nonlinear gradient denoising: Finding accurate extrema from inaccurate functional derivatives. *Int. J. Quantum Chem.* **115**, 1102–1114 (2015).

[8] Schölkopf, B., Smola, A. & Müller, K.-R. Nonlinear component analysis as a kernel eigenvalue problem. *Neural Comput.* **10**, 1299–1319 (1998).

[9] Király, F. J., Kreuzer, M. & Theran, L. Learning with cross-kernels and ideal PCA (2014). URL `http://arxiv.org/abs/1406.2646`.

[10] Schölkopf, B., Mika, S., Burges, C., Knirsch, P., Müller, K.-R., Rätsch, G. & Smola, A. Input space versus feature space in kernel-based methods. *IEEE Trans. Neural Netw.* **10**, 1000–1017 (1999).

[11] Giannozzi, P. *et al.* Quantum espresso: a modular and open-source software project for quantum simulations of materials. *J. Phys. Condens. Matter* **21**, 395502 (19pp) (2009).

[12] Bahn, S. R. & Jacobsen, K. W. An object-oriented scripting interface to a legacy electronic structure code. *Comput. Sci. Eng.* **4**, 56–66 (2002).

[13] Steinhaus, H. Sur la division des corps matériels en parties. *Bull. Acad. Polon. Sci. Cl. III.* **4**, 801–804 (1957) (1956).

[14] Hohenberg, P. & Kohn, W. Inhomogeneous electron gas. *Phys. Rev.* **136**, B864–B871 (1964).

[15] Kohn, W. & Sham, L. J. Self-consistent equations including exchange and correlation effects. *Phys. Rev.* **140**, A1133–A1138 (1965).

[16] Pribram-Jones, A., Gross, D. A. & Burke, K. DFT: A theory full of holes? *Annu. Rev. Phys. Chem.* **66**, 283–304 (2015).
